# Characteristics of Nursing Home Resident Movement Patterns: Results from the TEAM-UP Trial

**DOI:** 10.1097/01.ASW.0000822696.67886.67

**Published:** 2022-04-13

**Authors:** Susan M. Kennerly, Phoebe D. Sharkey, Susan D. Horn, Tianyu Zheng, Jenny Alderden, Valerie K. Sabol, Meredeth Rowe, Tracey L. Yap

**Affiliations:** Susan M. Kennerly, PhD, RN, CNE, WCC, FAAN, is Professor, College of Nursing, East Carolina University, Greenville, North Carolina, United States. Phoebe D. Sharkey, PhD, is Professor Emerita, Sellinger School of Business, Loyola University Maryland, Baltimore, Maryland. Susan D. Horn, PhD, is Adjunct Professor, School of Medicine, University of Utah, Salt Lake City. Tianyu Zheng, MS, is Biostatistician, Department of Population Health Sciences, University of Utah. Jenny Alderden, PhD, APRN, is Associate Professor, School of Nursing, Boise State University, Boise, Idaho. Valerie K. Sabol, PhD, ACNP, GNP, CNE, ANEF, FAANP, FAAN, is Professor, School of Nursing, Duke University, Durham, North Carolina. Meredeth Rowe, PhD, RN, FGSA, FAAN, is Professor, College of Nursing, University of South Florida Health, Tampa. Tracey L. Yap, PhD, RN, CNE, WCC, FGSA, FAAN, is Associate Professor, School of Nursing, Duke University.

**Keywords:** dementia, movement, nursing home, obesity, positioning, pressure injury, repositioning TEAM-UP

## Abstract

**OBJECTIVE:**

To determine movement patterns of nursing home residents, specifically those with dementia or obesity, to improve repositioning approaches to pressure injury (PrI) prevention.

**METHODS:**

A descriptive exploratory study was conducted using secondary data from the Turn Everyone And Move for Ulcer Prevention (TEAM-UP) clinical trial examining PrI prevention repositioning intervals. K-means cluster analysis used the average of each resident’s multiple days’ observations of four summary mean daily variables to create homogeneous movement pattern clusters. Growth mixture models examined movement pattern changes over time. Logistic regression analyses predicted resident and nursing home cluster group membership.

**RESULTS:**

Three optimal clusters partitioned 913 residents into mutually exclusive groups with significantly different upright and lying patterns. The models indicated stable movement pattern trajectories across the 28-day intervention period. Cluster profiles were not differentiated by residents with dementia (n = 450) or obesity (n = 285) diagnosis; significant cluster differences were associated with age and Braden Scale total scores or risk categories. Within clusters 2 and 3, residents with dementia were older (*P* < .0001) and, in cluster 2, were also at greater PrI risk (*P* < .0001) compared with residents with obesity; neither group differed in cluster 1.

**CONCLUSIONS:**

Study results determined three movement pattern clusters and advanced understanding of the effects of dementia and obesity on movement with the potential to improve repositioning protocols for more effective PrI prevention. Lying and upright position frequencies and durations provide foundational knowledge to support tailoring of PrI prevention interventions despite few significant differences in repositioning patterns for residents with dementia or obesity.

## INTRODUCTION

Nursing home (NH) residents are often advanced in age, cognitively challenged, and overweight or obese, making limitations in residents’ mobility/movements a common and increasing concern. Lack of movement for prolonged periods without relieving pressure on tissues compressed between the skin and support surface can lead to tissue damage, necrosis, and even pressure injuries (PrIs).^1^ In this study, the authors explore whether distinct movement patterns of NH residents can be identified using triaxial accelerometer data to ultimately advance knowledge related to repositioning approaches for PrI prevention. Movement associated with repositioning to prevent or relieve pressure is a primary pillar of prevention. However, little is known about how frequently an NH resident changes position or how long a single position is typically maintained. Characteristics of movement patterns for residents with dementia or obesity are also not well understood. Enhancing understanding about these movement patterns, especially among residents with conditions such as cognitive impairment and obesity, which are known to interfere with or limit movement, can provide insights about specific repositioning care needs. Discovery of differences could make it possible to tailor and test resident-focused PrI prevention interventions, such as repositioning, and help residents with dementia and obesity adopt movement behaviors that are effective in reducing PrI development.

At least one of every nine NH residents in the US experiences a PrI at some point during their NH stay.^2,3^ Incidence of PrIs increased by 10% nationwide between 2014 and 2016 despite reductions in most other hospital-acquired conditions.^4^ Many NHs have high PrI incidence and prevalence, in some instances well over 20%.^5,6^ A PrI can develop when repositioning/movement is inadequate, exposing an area of tissue to high levels of constant pressure, which, if unrelieved, reduces blood flow and prevents tissue reperfusion, leading to tissue necrosis.^7^ After prolonged pressure on tissues, such as when a resident lies in the same position for an extended period or has limited mobility, a minimum of 15 minutes’ decompression time is required for adequate reperfusion of ischemic tissues. Prevention is commonly achieved through vigilant resident repositioning (every 2 hours), which requires labor-intensive nursing care; repeated pressure exposure aggravates tissues, making them more vulnerable with each subsequent pressure-loading event. For example, a resident who is repositioned but slides back into the original position before 15 minutes has elapsed may have inadequate tissue offloading, which can lead to additional insult. The combination of pressure, time, and cycles of tissue ischemia-reperfusion that results in PrIs varies widely among residents. Evidence from prior research is clear that the process of damage accumulation in the tissues occurs when a repositioning event does not completely offload pressure between the tissue and external support surface.^8^ Movement patterns are critical to understanding PrI prevention. Also, movements are associated with friction and shear that occur in varied stages of dementia and obesity.

Historically, nearly half of NH residents have Alzheimer disease and related dementias (hereafter, dementia),^9^ and 25% of all NH residents are classified as obese.^10^ Crucial to improving quality of life for persons with dementia living in an NH is forestalling acute medical problems that are mainly preventable, seemingly intractable geriatric conditions, such as PrI development. Dementias increase with age, and typically an individual with dementia is unsteady and slow to move about. Obesity also limits the capacity for movement and is considered a risk factor in PrI development. In fact, persons with a high body mass index (BMI) who also have limited mobility are more likely to develop dementia.^10–12^ The average BMI of the population is increasing; according to VanGilder et al,^13^ average height and weight increased by 1 inch and 23 to 25 lb, respectively, over the past 4 to 5 decades. Older adults with obesity are twice as likely as those who are nonobese (BMI ≤30 kg/m^2^) to be admitted to an NH and often experience more disabilities that require more complex care.^14^ Dementia and obesity are thought to contribute to reduced mobility, putting residents at risk of developing a PrI, and the cornerstone of prevention care is self- or nurse-assisted repositioning/movement that aims to facilitate the ≥15-minute decompression time^15^ that is required for tissue reperfusion. The etiology of PrI development is largely dependent on intensity and duration of pressure; however, there is a gap in evidence related to the duration underpinning the care approach to PrI prevention, especially when the care approach is tailored to residents with dementia and obesity.

Dementia and obesity are increasingly prevalent factors among NH residents and are becoming more clinically relevant because of the potential increase in PrI risk.^10,16–18^ Further, these two factors often limit residents’ ability to participate in their own care, impair mobility, and are associated with prolonged periods in either a bed or chair, which increases the interface pressure between the body and supporting surface. A first step in understanding how dementia and obesity relate to residents’ mobility is to determine what movement patterns exist among all residents.

## METHODS

### Aims

The primary aim of this study was to identify and characterize types and frequencies of position changes comprising movement patterns for NH residents. The authors explored resident characteristics (age, sex, race/ethnicity, average Braden Scale total score, Braden Scale risk categories, history of PrI, dementia and/or obesity diagnosis, and NHs) as potential predictors of movement patterns based on position frequencies and durations.

### Design

This descriptive exploratory study was conducted using secondary data collected in nine NHs in the 1R01NR016001 Turn Everyone And Move for Ulcer Prevention (TEAM-UP) cluster randomized controlled trial.^19,20^ The TEAM-UP trial examined the effects of repositioning frequency on PrI incidence using an NH-wide protocol with repositioning intervals of 2, 3, or 4 hours on residents at low, mild, moderate, or high risk of developing PrIs over a 4-week intervention period. Each NH was randomly assigned to one of three study arms (2, 3, or 4 hours) while continuing to provide standard PrI prevention and other nursing care. Residents’ repositioning movements were tracked in real time with a triaxial accelerometer wireless sensor monitoring system (MS) that recorded all resident movement (except for those residents with existing PrIs or at severe PrI risk). Residents’ movements were collected during a 4-week intervention period; the number of days of movement data varied based on resident length of stay. The target sample of the TEAM-UP study was powered at 95% with a one-tailed significance level of .05 to detect an effect size of .38 to test an increase in PrI incidence from standard 2-hour repositioning to 3 or 4 hours.^19,20^

### Study Setting and Population Sample

The sample of residents from all nine NH sites (N = 913) included residents with dementia and/or obesity as part of the population examined in the secondary data analyses. The TEAM-UP trial enrollment, allocation, and follow-up are reported elsewhere.^19,20^ All residents participating in the TEAM-UP study’s intervention were eligible for inclusion without regard to diagnoses or demographic characteristics, which were subsequently analyzed.

Dementia is a descriptive term used to describe those in the sample with a syndrome of degenerative brain changes characterized by a set of symptoms, including cognitive and behavioral symptoms, which are described as behavioral and psychological symptoms of dementia; Alzheimer disease is the most common cause of dementia. Dementia is often formally diagnosed in the later stages of disease. In this study, residents were identified as having dementia if there was an *International Classification of Diseases* diagnosis of dementia according to the Coding Definition provided by the National Institutes of Health^21^ and/or a Brief Interview for Mental Status (BIMS) score indicative of cognitive impairment (severe impairment = 0–7, moderate impairment = 8–12). Often, there is no formal diagnosis given if a resident develops dementia after admission, thus the addition of BIMS.

Obesity is defined for this sample as a body weight that is higher than what is considered to be a healthy weight when compared with height.^22^ Body mass index (BMI) is most often used to determine whether an individual is underweight (BMI <18.5 kg/m^2^), normal weight (BMI 18.5 to <25 kg/m^2^), overweight (BMI 25–30 kg/m^2^), or obese (BMI >30 kg/m^2^) and is calculated by dividing a person’s weight in kilograms by the square of height in meters.^22^ In this study, obesity was categorized as BMI >30 kg/m^2^.

### Measurement

#### Clinical Measures

The Braden Scale for Predicting Pressure Sore Risk^23^ was used to determine PrI risk status. This summed rating scale is composed of six subscales (sensory perception, mobility, activity, moisture, nutrition, and friction and shear) and was developed to help clinicians predict risk for PrI development and to guide preventive measures based on risk factors. The six subscales are rated from 1 to 4 (except friction and shear, which is rated from 1–3), with 6 to 23 total points possible. The cutoff for diagnosing risk is ≤18.^24^ The tool’s predictive validity varies by setting, but it is the most accurate available measure. Admission overall sensitivity (74%) and specificity (60%) are not as accurate as observations made 24 hours after admission, when sensitivity is 76% and specificity is 68%. Weekly/monthly observations are even more accurate, increasing to 81% sensitivity and 73% specificity.^24,25^ Risk categories are based on Braden Scale total scores: low (19–23), mild (15–18), moderate (13–14), and high (10–12) risk.

####  Movement Metrics

Movement is defined as all active or passive movements that were tracked and recorded every 10 seconds by the MS sensor from the start to end of a resident’s TEAM-UP study participation for each day in which a complete record of 22 to 24 hours was collected.

#####  Movement pattern

The naturally and most frequently occurring distribution of movement features (body position frequency and position durations, lying and upright movement frequencies and durations).

#####  Body position frequency

Body position was the direction and angle in which a person was facing lying in bed (left, left prone, right, right prone, back) or upright when sitting in bed or a chair (back, left, right). Threshold parameters for position detection are 10-degree tilt angle (leaning side-to-side when upright), 50-degree upright angle (sitting versus lying), and 20-degree turn angle (change from one side to another). Frequency equaled the number of times a specific direction and angle were achieved in 24 hours. Upright time while walking was not included because the focus of this study is on understanding offloading in bed or chair.

#####  Body position duration

The sum of the length of all time per day spent in a single body position.

#####  Lying and upright movement frequencies

The sum of body position frequencies while in lying or upright position in 24 hours. These frequencies reflect the daily number of times a repositioning event occurs while either lying or upright (ie, the resident engages in movement or is assisted by nursing staff to move).

#####  Lying and upright movement durations

The sum of body position durations while in lying or upright positions in 24 hours; this reflects the total daily time in lying or upright positions.

####  Data and Data Management

##### Nursing Home Data

The NH characteristics collected in the TEAM-UP study included bed size and nursing staff hours, which were provided by the NH company and collected from publicly available sources.^26^

#####  Resident Electronic Health Record (EHR) Data

These data for each resident participating in the intervention were extracted from the EHR for each week of the study period according to the TEAM-UP protocol. All EHR data extractions were performed by the NH company in a Health Insurance Portability and Accountability Act-compliant format; study identification numbers (study IDs) were created prior to data extraction, and data were transferred to Duke University’s secure drive space designated for the TEAM-UP study, from which this study accessed EHR data for secondary analysis.

#####  Resident movement data

The wireless sensor worn by each resident participating in the intervention communicated directly with the MS database housed on a secure remote server. Resident movement data were collected every 10 seconds, 24 hours a day while a sensor was worn. The MS database also received the resident’s study ID along with admission, transfer, and discharge status updates in real time. The MS raw data files and summarized data by resident, unit, and NH were transferred to Duke University’s secure drive space designated for the TEAM-UP study, from which this study accessed movement data for secondary analysis. Fidelity checks were used to ensure data trustworthiness, including observation audits to safeguard proper implementation such as on-time turning.

### Analysis

#### Sensor Data Observations

Resident wearable sensors recorded the start and stop times for every individual movement change. These recordings were summarized by the distinctive movement positions measuring daily frequencies and durations for lying and upright left, right, and back body positions. The average daily movement frequencies and durations were explored initially to identify resident movement behaviors and develop groups of homogeneous patterns. Subsequently, these data were collapsed into four summary variables: total daily upright time, total daily upright frequency, total daily lying time, and total daily lying frequency. Because there were days when the sensor recording was interrupted due to resident off-site visits or discharge, any resident days’ observations that were less than 22 out of 24 hours were omitted.

####  Statistical Analysis

Cluster analysis used the mean of each resident’s multiple days’ observations of the four summary total daily variables (lying and upright movement frequencies, lying and upright movement durations) to create homogeneous movement pattern clusters. K-means cluster analyses were used to identify an unknown number (“*k*”) of clusters with distinct, interpretable, and/or usable movement patterns. The first step in identifying these movement patterns was to group together residents with relatively homogeneous average daily movement features. The researchers used the cubic clustering criterion to determine optimal clusters that partitioned residents into mutually exclusive groups based on their four input variables. A good clustering algorithm aims to obtain clusters where (1) the intracluster similarities are high, implying that the residents in a cluster are similar to each other in terms of their movement features; and (2) the intercluster similarity is low, implying that each cluster contains residents whose movement features are not similar to those of residents in other clusters.^27^

In addition, longitudinal analyses of the data were conducted using growth mixture modeling (GMM) to examine and confirm the extent of any changes in an individual’s daily movement patterns over time. Growth mixture modeling describes longitudinal change across the intervention time period within each individual in each cluster and examines if differences in individual patterns exist within a cluster of study participants. As an extension of individual growth curve analysis, GMM models longitudinal trajectories of measures at an individual level. However, GMM has the additional advantage of identifying distinct classes of movement pattern trajectories rather than assuming the same mean trajectory over time for all residents within the same group.

Descriptive statistics (means, SDs, percentages) were used to describe resident demographic and clinical characteristics by individual NH and movement pattern cluster. Logistic regression analyses were conducted to predict resident and NH membership in the cluster groups. Predictors included age, sex, diagnoses of dementia and/or obesity, history of previously healed PrI, race, Braden Scale risk categories, and NHs. The researchers performed separate analyses for the resident populations with dementia and with obesity to evaluate differences within and between these populations by cluster. Comparisons among NHs, cluster groups, and the dementia and obesity populations were made using *χ*^2^, analysis of variance, or two-sample *t* tests.

### Ethics Approval and Consent to Participate

The TEAM-UP project was approved by the Duke University Institutional Review Board (Duke IRB #Pro00069413). The IRB approved a waiver of informed consent per the US Department of Health and Human Services guidelines 21 CFR 46 because (1) the entire group of low-, moderate-, and high-risk residents received an NH-wide repositioning schedule; (2) repositioning protocol became part of NH-wide practice standardizing the workflow of repositioning; (3) the intervention involved minimal risk; and (4) extracted resident data were assigned a study ID number and the coded data set was placed directly into a secured network folder. For the Administrative Supplement involving cluster analysis, per the US Department of Health and Human Services guidelines 21 CFR 46, the Duke IRB waived informed consent for the NH residents and approved staff consent to participate (Duke IRB #Pro00069413). All methods were performed in accordance with the relevant guidelines and regulations (Declaration of Helsinki).

## RESULTS

Intervention residents with 1 or more days with 22 to 24 hours of sensor observations were included in the analyses; 913 of 1,100 residents in the intervention study met this criterion. K-means did not generate the optimal number of clusters, thereby requiring an exploratory approach to predetermine a value of *k* that produced effective results. Multiple cluster solutions were evaluated with plotting of the cubic clustering criterion for each value of *k*. Values for *k* ≥ 3 indicated good results. However, additional splits above three produced clusters with small numbers of observations that were not large enough to warrant special treatment. The value *k* = 3 was chosen as the most useful partitioning of the data for this study. The GMM models analyzing the movement pattern trajectories across the 28-day intervention period were stable, indicating that the cluster analysis groups based on the variable means over time for all residents were an accurate indicator of their behaviors. The decision to accept the three clusters produced by K-means cluster analysis was made by the principal investigators and content collaborators.

Characteristics of the 913 study residents are presented in Table 1, both overall and for each of the nine NHs. All resident characteristics were significantly different across NHs. In particular, NH4 and NH9 had a greater number and percent of Black residents, NH4 had a greater number of residents with previously healed PrIs, and NH7 had a higher number of movements per hour during lying and upright times. In total, 5.26% (48/913) of residents had a history of healed PrIs. Overall, the population studied comprised 50% individuals with dementia and 31% individuals with obesity, which are similar percentages to those typically found in NHs.

**Table 1:** RESIDENT CHARACTERISTICS BY NH

Characteristics	Total N = 913	NH1 n = 65	NH2 n = 107	NH3 n = 103	NH4 n = 105	NH5 n = 113	NH6 n = 129	NH7 n = 90	NH8 n = 84	NH9 n = 117	*P*
Age, mean (SD)	77.7 (3.0)	76.4 (13.2)	79.3 (12.7)	86.8 (8.7)	81.3 (12.3)	72.4 (12.3)	74.3 (12.7)	75.9 (12.9)	79.2 (12.4)	75.0 (13.9)	<.0001^a^
Sex, male, n (%)	349 (38.2)	26 (40.0)	35 (32.7)	20 (19.4)	41 (39.1)	56 (49.6)	44 (34.1)	37 (41.1)	31 (36.9)	59 (50.4)	<.0001^a^
Race											
Black, n (%)	260 (28.5)	7 (10.8)	6 (5.6)	0	70 (66.7)	22 (19.5)	28 (21.7)	15 (16.7)	15 (17.9)	97 (82.9)	
White, n (%)	508 (65.5)	52 (80.0)	93 (86.9)	9 (8.7)	32 (30.5)	79 (69.9)	101 (78.3)	71 (78.9)	61 (72.6)	15 (12.8)	<.0001^b^
Other, n (%)	55 (6.02)	6 (9.2)	8 (7.5)	94 (91.3)	3 (2.9)	12 (10.6)	0	4 (4.4)	8 (9.5)	5 (4.3)	
History of prior PrI, healed, n (%)	48 (5.26)	2 (3.1)	5 (4.7)	2 (1.9)	15 (14.3)	7 (6.2)	4 (3.1)	4 (4.4)	7 (8.3)	2 (1.7)	.001^b^
ADRD, n (%)	450 (49.3)	43 (66.2)	51 (47.7)	28 (27.2)	65 (61.9)	43 (38.1)	47 (36.4)	51 (56.7)	55 (65.5)	67 (57.3)	<.0001^b^
Obesity, n (%)	285 (31.2)	19 (29.2)	37 (34.6)	27 (26.2)	19 (18.1)	31 (27.4)	52 (40.3)	32 (35.6)	33 (39.3)	35 (29.9)	.0126^b^
ADRD no/obese no, n (%)	298 (32.6)	13 (20.0)	37 (34.6)	57 (55.3)	30 (28.6)	48 (42.5)	47 (36.4)	20 (22.2)	13 (15.5)	33 (28.2)	
ADRD no/obese yes, n (%)	165 (18.1)	9 (13.9)	19 (17.8)	18 (17.5)	10 (9.5)	22 (19.5)	35 (27.1)	19 (21.1)	16 (19.1)	17 (14.5)	<.0001^b^
ADRD yes/obese no, n (%)	330 (36.1)	33 (50.8)	33 (30.8)	19 (18.5)	56 (53.3)	34 (30.1)	30 (23.3)	38 (42.2)	38 (45.2)	49 (41.9)	
ADRD yes/obese yes, n (%)	120 (13.1)	10 (15.4)	18 (16.8)	9 (8.7)	9 (8.6)	9 (8.0)	17 (13.2)	13 (14.4)	17 (20.2)	18 (15.4)	
Lying freq/hour, mean (SD)	7.6 (8.1)	6.0 (3.5)	6.7 (4.9)	5.4 (5.1)	7.8 (6.6)	7.5 (6.2)	6.4 (4.0)	16.9 (18.4)	6.2 (5.6)	6.0 (3.7)	<.0001^a^
Upright freq/hour, mean (SD)	30.7 (32.2)	25.3 (20.7)	33.7 (48.3)	22.7 (15.9)	27.8 (17.8)	30.6 (16.8)	31.4 (22.5)	45.2 (52.7)	32.0 (38.7)	28.1 (32.9)	.0305^a^
Total lying hours/day, mean (SD)	14.96 (4.4)	14.8 (4.4)	14.7 (4.2)	13.1 (3.2)	14.1 (4.3)	14.7 (4.5)	15.6 (4.3)	16.2 (5.0)	14.6 (4.0)	16.6 (4.6)	<.0001^a^
Total upright hours/day, mean (SD)	7.96 (4.35)	8.2 (4.5)	8.0 (4.0)	9.6 (3.2)	8.3 (4.1)	8.2 (4.4)	7.5 (4.3)	6.9 (5.0)	8.7 (4.1)	6.7 (4.7)	<.0001^a^

Abbreviations: ADRD, Alzheimer disease and related dementias; NH, nursing home; PrI, pressure injury.

^a^Analysis of variance used to test for mean differences across NHs.

^b^*χ*^2^ Test used to test for frequency differences across NHs.

Cluster analysis categorized groups of residents according to their movement features. Table 2 presents data describing features of the three generated clusters. Cluster 1, the smallest cluster, contained 52 residents (5.7%) who spent approximately 18.6 hours lying and 4.7 hours upright with very high frequency of lying (20.2) and upright (52.8) movements per hour. Cluster 2 contained 378 residents (41.4%) with similar lying and upright hours to cluster 1, but much lower frequency of lying (5.2) and upright (29.5) movements per hour. Thus, residents in cluster 1 moved approximately four times more frequently when lying and approximately two times more frequently when upright than did residents in cluster 2. Cluster 3 contained the majority of the population, comprising 483 residents (52.9%) with almost equal time spent lying (11.6 hours) and upright (11.3 hours). These residents’ frequencies of movements per hour were 8.1 (lying) and 29.2 (upright).

**Table 2:** CLUSTER CHARACTERISTICS (N = 913)

Characteristic	Cluster 1 (n = 52) Mean (SD)	Cluster 2 (n = 378) Mean (SD)	Cluster 3 (n = 483) Mean (SD)	*P*
Lying frequency/hour	20.2 (8.4)	5.2 (2.6)	8.1 (9.5)	<.0001^a^
Upright frequency/hour	52.8 (33.7)	29.5 (45.2)	29.2 (14.5)	<.0001^a^
Total lying hours/day	18.6 (3.0)	18.8 (2.7)	11.6 (2.5)	<.0001^b^
Total upright hours/day	4.7 (3.0)	4.2 (2.6)	11.3 (2.5)	<.0001^b^
Minutes in each lying body position	3.39 (1.1)	15.04 (8.7)	12.10 (8.5)	<.0001^b^
Minutes in each upright body position	1.60 (1.1)	5.54 (7.3)	2.96 (3.2)	<.0001^b^

^a^*χ*^2^ Test used to test for frequency differences across clusters.

^b^Analysis of variance used to test for mean differences across clusters.

Clusters were differentiated by movement frequencies associated with five lying (left, left prone, right, right prone, back) and three upright (back, left, right) body position changes. When lying, cluster 1 spent an average of 3.39 minutes per single position, whereas cluster 2 spent an average of 15.04 minutes per single position (*P* < .001). These two clusters were also differentiated by minutes of three upright body position changes, resulting in cluster 1 having an average of 1.60 minutes per position versus cluster 2 having an average of 5.54 minutes per position (*P* < .001). Cluster 3 had an average of 12.10 minutes per position lying and 2.96 minutes per position upright.

Resident and NH characteristics associated with membership in each cluster are presented in Table 3. Membership in cluster 1 was only predicted by residence in NH7 (odds ratio [OR], 10.15; confidence interval [CI], 5.43–19.37). Membership in cluster 2 was predicted by greater likelihood of being in Braden Scale risk categories of mild (OR, 2.58; CI, 1.83–3.64), moderate (OR, 10.68; CI, 6.41–17.80), and high (OR, 13.48; CI, 6.70-27.12) compared with being in the low-risk category reference group, and lower likelihood of being in NH1, NH3, NH5, NH7, or NH8 compared with being in NH9 (reference group, *P* < .05, tabled). Membership in cluster 3 was predicted by lower likelihood of being in Braden Scale risk categories of mild (OR, 0.37; CI, 0.27–0.52), moderate (OR, 0.09; CI, 0.06-0.16), and high (OR, 0.07; CI, 0.03–0.15) compared with being in the low-risk category, and greater likelihood of being in NH3 and NH8 compared with being in NH9 (reference group, *P* < .05, tabled). The three clusters were not differentiated by dementia or obesity resident diagnoses.

**Table 3:** ODDS RATIOS AND CONFIDENCE INTERVALS FOR PREDICTORS OF BEING IN EACH CLUSTER (N = 913)

	Cluster 1, OR (CI), n = 52	Cluster 2, OR (CI), n = 378	Cluster 3, OR (CI), n = 483
ADRD	1.35 (0.71–2.56)	0.95 (0.69–1.30)	1.00 (0.73–1.37)
Obesity	0.94 (0.48–1.83)	0.98 (0.70–1.35)	1.04 (0.76–1.44)
Race: Black	0.69 (0.32–1.46)	0.95 (0.63–1.43)	1.12 (0.75–1.68)
Race: other	0.28 (0.04–2.19)	0.79 (0.41–1.51)	1.52 (0.80–2.88)
History of prior pressure injury, healed	NA^a^	1.65 (0.85–3.22)	0.83 (0.43–1.62)
Braden mild risk	1.49 (0.72–3.10)	2.58 (1.83–3.64)^b^	0.37 (0.27–0.52)^b^
Braden moderate risk	0.96 (0.34–2.70)	10.68 (6.41–17.80)^b^	0.09 (0.06–0.16)^b^
Braden high risk	1.16 (0.34–3.92)	13.48 (6.70–27.12)^b^	0.07 (0.03–0.15)^b^
Age	0.99 (0.97–1.02)	0.98 (0.96–0.99)^c^	1.02 (1.01–1.04)^c^
Male sex	1.08 (0.57–2.04)	1.23 (0.90–1.69)	0.81 (0.59–1.10)
NH1	NA	0.46 (0.22–0.96)^d^	1.70 (0.82–3.50)
NH2	NA	0.52 (0.27–1.00)	1.87 (0.98–3.58)
NH3	NA	0.44 (0.22–0.88)^d^	2.24 (1.12–4.45)^d^
NH4	NA	0.65 (0.36–1.17)	1.32 (0.73–2.36)
NH5	NA	0.47 (0.25–0.88)^d^	1.72 (0.92–3.20)
NH6	NA	0.82 (0.45–1.50)	1.08 (0.59–1.98)
NH7	10.15 (5.43–19.37)^b^	0.23 (0.12–0.46)^b^	1.22 (0.63–2.37)
NH8	NA	0.26 (0.13–0.53)^c^	3.31 (1.65–6.67)^c^
C statistic	0.76	0.74	0.74

Abbreviations: ADRD, Alzheimer disease and related dementias; CI, confidence interval; NA, not applicable; NH, nursing home; OR, odds ratio.

NA: Because of small sample size in cluster 1, a two-step process produced one NH as the only significant predictor. Reference groups include White race, Braden Scale low risk, female, non-ADRD, nonobese, and NH9.

^a^Zero previously healed pressure injuries in cluster 1.

^b^*P* < .0001.

^c^*P* < .001.

^d^*P* < .05.

Characteristics examined separately within resident cohorts with dementia and obesity across each of the three clusters are described in Table 4. The three clusters differed significantly by age for residents with dementia (*P* = .0004) but not for residents with obesity, by race for residents with obesity (*P* = .0051) but not for those with dementia, and by mean Braden Scale total scores (*P* < .0001) and risk categories (*P* < .0001) within both resident cohorts. Overall, 5.8% (26/450) of residents with dementia had a healed PrI compared with only 2.8% (8/285) of residents with obesity.

**Table 4:** RESIDENTS’ CHARACTERISTICS BY CLUSTER ACCORDING TO DEMENTIA AND OBESITY CATEGORIES (N = 913)

Characteristic	Residents with Dementia (n = 450)				Residents with Obesity (n = 285)			
	Cluster 1 (n = 52)	Cluster 2 (n = 378)	Cluster 3 (n = 483)	*P* (ANOVA/*χ***^2^**)	Cluster 1 (n = 52)	Cluster 2 (n = 378)	Cluster 3 (n = 483)	*P* (ANOVA/*χ***^2^**)
n (%) in cluster	30 (57.7)	189 (50)	231 (47.8)	—	17 (32.70)	113 (29.9)	155 (32.1)	—
Age, mean (SD), y	80.0 (9.9)	77.9 (12.9)	82.4 (10.5)	.0004	75.2 (12.4)	71.7 (13.4)	74.5 (13.0)	.1974^a^
Male, n (%)	11 (36.7)	77 (40.7)	84 (36.4)	.6452^b^	5 (29.4)	41 (36.3)	61 (39.4)	.6795^b^
Race, n (%)								
Black	5 (16.7)	73 (38.6)	70 (30.3)	.1084^b^	3 (17.7)	42 (37.2)	28 (18.1)	.0051^b^
White	24 (80.0)	108 (57.1)	149 (64.5)		13 (76.5)	68 (60.2)	115 (74.2)	
Other	1 (3.3)	8 (4.2)	12 (5.2)		1 (5.9)	3 (2.7)	12 (7.7)	
History of healed PrI, n (%)	0	12 (6.4)	14 (6.1)	.3703^b^	0	5 (4.4)	3 (1.9)	.3667^b^
Obesity, n (%)	9 (30.0)	49 (25.9)	62 (26.8)	.8927^b^	17 (100)	113 (100)	155 (100)	NA
Dementia, n (%)	30 (100)	189 (100)	231 (100)	NA	9 (52.9)	49 (43.4)	62 (40.0)	.556^b^
Lying freq/hour, mean (SD)	21.3 (8.7)	5.3 (2.7)	8.0 (11.9)	<.0001^a^	19.0 (6.7)	5.3 (2.7)	8.3 (13.3)	<.0001^a^
Upright freq/hour, mean (SD)	54.7 (39.9)	29.1 (51.0)	27.8 (14.0)	<.0001^a^	48.3 (21.2)	32.9 (43.2)	31.6 (15.5)	<.0001^a^
Total lying hours/day, mean (SD)	18.1 (3.1)	18.9 (2.7)	11.6 (2.6)	<.0001^a^	18.4 (2.1)	19.0 (2.4)	11.7 (2.6)	<.0001^a^
Total upright hours/day, mean (SD)	5.2 (3.2)	4.1 (2.6)	11.3 (2.5)	<.0001^a^	4.9 (2.1)	3.9 (2.4)	11.2 (2.6)	<.0001^a^
Braden Scale score, mean (SD)	16.5 (3.1)	15.7 (2.8)	18.2 (2.6)	<.0001^a^	17.0 (2.6)	17.2 (3.1)	18.7 (2.5)	<.0001^a^
Braden Scale risk categories								
Low risk, n (Col %)	7 (23.3)	25 (13.2)	99 (42.9)	<.0001^b^	3 (17.7)	30 (26.6)	78 (50.3)	< .0001^b^
Mild risk, n (Col %)	13 (43.4)	86 (45.5)	109 (47.2)		11 (64.7)	57 (50.4)	69 (44.5)	
Moderate risk, n (Col %)	6 (20)	58 (30.7)	18 (7.8)		1 (5.9)	19 (16.8)	6 (3.9)	
High risk, n (Col %)	4 (13.3)	20 (10.6)	5 (2.2)		2 (11.8)	7 (6.2)	2 (1.3)	

Abbreviations: ANOVA, analysis of variance; NA, not applicable; PrI, pressure injury.

^a^ANOVA used to test for mean differences among clusters.

^b^*χ*^2^ Test used to test for frequency differences among clusters.

Movement patterns were found to differ between these resident cohorts as well. The means of lying time, lying frequency, upright time, and upright frequency across the clusters (Table 4) revealed that cluster 1 is dominated by an average of 18.1 (dementia) to 18.4 (obesity) hours of lying time per day with an average of 19.0 to 21.3 lying frequency changes per hour. Cluster 2 is also dominated by an average of 18.9 to 19.0 hours of lying time per day but had only 5.3 average hourly lying frequency changes. Cluster 3 had relatively equal amounts of average lying and upright times (11.6–11.7 and 11.2–11.3 hours, respectively) and frequency changes per hour (8.0–8.3 lying and 27.8–31.6 upright). The three clusters differed significantly within the populations with dementia and obesity for all four frequency and duration measures (*P* < .0001).

Comparisons of resident characteristics between individuals with dementia and those with obesity within each cluster are presented in Table 5. Residents with dementia were significantly older than residents with obesity in clusters 2 and 3 (*P* < .0001). Race differences between the two populations existed only in cluster 3, where significantly more residents in the dementia cohort were Black (*P* = .005) and significantly more residents with obesity were White (*P* = .045). Across all NHs, there were 260 Black residents: 73 with obesity, 148 with dementia, 36 who had both diagnoses, and 75 who had neither diagnosis. The population of Black residents with obesity was predominately in cluster 2 (37.2%) compared with 17.7% in cluster 1 and 18.1% in cluster 3. The population of Black residents with dementia was also predominately in cluster 2 (38.6%) compared with 16.7% in cluster 1 and 30.3% in cluster 3. Cluster 3 residents with dementia had a three times higher percentage rate (*P* = .033) of healed PrIs (6.1%) compared with cluster 3 residents with obesity (1.9%). The PrI history demonstrated a higher risk of developing a PrI among residents with dementia (5.77% [26/450]) than those with obesity (2.81% [8/285]; Table 4). Braden Scale mean scores were significantly worse in cluster 2 (*P* < .0001) for the population with dementia, which had fewer low-risk and more moderate-risk residents compared with residents with obesity. There were no significant differences by cluster between residents with dementia and residents with obesity for total lying frequency per hour, total lying hours per day, or total upright hours per day. However, total upright frequency per hour in cluster 2 was significantly higher for residents with obesity compared with residents with dementia (*P* = .0352).

**Table 5:** RESIDENTS’ CHARACTERISTICS WITHIN CLUSTER BETWEEN DEMENTIA AND OBESITY CATEGORIES (N = 913)

Characteristic	Cluster 1 (n = 52)			Cluster 2 (n = 378)			Cluster 3 (n = 483)		
	Dementia	Obesity	*P*	Dementia	Obesity	*P*	Dementia	Obesity	*P*
n (%) in cluster	39 (57.7%)	17 (32.70%)	-	189 (50%)	113 (29.9%)	-	231 (47.8%)	155 (32.1%)	-
Age, mean (SD)	80.0 (9.9)	75.2 (12.4)	.1497^a^	77.9 (12.9)	71.7 (13.4)	< .0001^a^	82.4 (10.5)	74.5 (13.0)	< .0001^a^
Male, n (%)	11 (36.7)	5 (29.4)	.6231^b^	77 (40.7)	41 (36.3)	.4440^b^	84 (36.4)	61 (39.4)	.5531^b^
Race, n (%)									
Black	5 (16.7)	3 (17.7)	.9334^b^	73 (38.6)	42 (37.2)	.8017^b^	70 (30.3)	28 (18.1)	.0050^b^
White	24 (80.0)	13(76.5)	.7821^b^	108 (57.1)	68 (60.2)	.6063^b^	149 (64.5)	115 (74.2)	.0448^b^
Other	1 (3.3)	1 (5.9)	.6843^b^	8 (4.2)	3 (2.7)	.4458^b^	12 (5.2)	12 (7.7)	.3288^b^
History of healed PrI, n (%)	0	0	0	12 (6.4)	5 (4.4)	.4842^b^	14 (6.1)	3 (1.9)	.0328^b^
Obesity, n (%)	9 (30.0)	17 (100)	NA	49 (25.9)	113 (100)	NA	62 (26.8)	155 (100)	NA
Dementia, n (%)	39 (100)	9 (52.9)	NA	189 (100)	49 (43.4)	NA	231(100)	62 (40.0)	NA
Lying freq/hour, mean (SD)	21.3 (8.7)	19.0 (6.7)	.5292^a^	5.3 (2.7)	5.3 (2.7)	.9884^a^	8.0 (11.9)	8.3 (13.3)	.5688^a^
Upright freq/hour, mean (SD)	54.7 (39.9)	48.3 (21.2)	.7140^a^	29.1 (51.0)	32.9 (43.2)	.0352^a^	27.8 (14.0)	31.6 (15.5)	.0636^a^
Total lying hours/day, mean (SD)	18.1 (3.1)	18.4 (2.1)	.7098^a^	18.9 (2.7)	19.0 (2.4)	.5595^a^	11.6 (2.6)	11.7 (2.6)	.5999^a^
Total upright hours/day, mean (SD)	5.2 (3.2)	4.9 (2.1)	.6895^a^	4.1 (2.6)	3.9 (2.4)	.5494^a^	11.3 (2.5)	11.2 (2.6)	.5381^a^
Braden Scale score, mean (SD)	16.5 (3.1)	17.0(2.6)	.5336^a^	15.7 (2.8)	17.2 (3.1)	<.0001^a^	18.2 (2.6)	18.7 (2.5)	.0539^a^
Braden Scale risk categories									
Low risk, n (Col %)	7 (23.3)	3 (17.7)	.6557^b^	25 (13.2)	30 (26.6)	.0066^b^	99 (42.9)	78 (50.3)	.1498^b^
Mild risk, n (Col %)	13 (43.4)	11 (64.7)	.1660^b^	86 (45.5)	57 (50.4)	.4071^b^	109 (47.2)	69 (44.5)	.6071^b^
Moderate risk, n (Col %)	6 (20)	1 (5.9)	.1432^b^	58 (30.7)	19 (16.8)	.0048^b^	18 (7.8)	6 (3.9)	.0965^b^
High risk, n (Col %)	4 (13.3)	2 (11.8)	.8802^b^	20 (10.6)	7 (6.2)	.1711^b^	5 (2.2)	2 (1.3)	.5089^b^

Abbreviations: PrI, pressure injury; SD, standard deviation.

^a^*T-*test used to test for differences in mean value data between dementia and obesity within the cluster.

^b^Chi square used to test for differences in count data between dementia and obesity within the cluster.

## DISCUSSION

This study is the first to demonstrate that there are major differences in movement patterns among NH residents. Identification of distinct movement patterns is essential for improving PrI prevention strategies, even though there may be more than three discoverable patterns that could be actionable. Analyses of the movement patterns in these clusters were based on duration of time spent in lying or upright positions and frequency of position changes, yielding clinically homogeneous subgroups of residents. It is noteworthy that the percentage of daily lying time was approximately 80% for clusters 1 and 2. Cluster 3 was unique because it had relatively equal lying (51%) and upright (49%) times. It is remarkable that residents in clusters 1 and 2 spent the overwhelming majority of the day in a lying position, yet residents did not remain lying in a single body position for more than, at most, an average of 15 minutes at a time. Despite the total amount of time spent lying per day, this finding suggests that periodically spending a shorter duration in lying in a single body position may be effective in offloading pressure points. This logic is supported by the absence of PrI development among residents with low, moderate, and high PrI risk in this clinical trial’s 4-week intervention period. Further, the relatively small amount of time spent in the same body position is believed to potentially support the appropriateness of the current standard^1^ of repositioning at-risk residents to offload tissues for at least 15 minutes. Additional research is needed to better understand how frequent and even slight position changes within an hour (sometimes called microturns) affect PrI prevention in contrast to a less frequent or complete change in body position.

The researchers explored the characteristics of residents that defined membership in the respective movement clusters to discern whether there were specific differences that may offer insights for clinical practice. For example, cluster 1 appears to be a small outlier population with the highest frequency of body position changes; cluster 1 characteristics could not be fully explored because of the small sample size. Detection of rare data where behavior is exceptional compared with the rest of a large data set can potentially lead to uncovering valuable knowledge hidden behind these observations.

Membership in clusters 2 and 3 is differentiated by residents’ Braden Scale risk categories. Odds ratios suggest that cluster 2 residents were approximately 13.5 times more likely to be in the Braden Scale high-risk category and 10.7 times more likely to be in the Braden Scale moderate-risk category compared with cluster 2 residents with low Braden Scale risk. Odds ratios confirm that residents with these same moderate or high Braden Scale risk categories are far less likely to be in cluster 3 (91% and 93% less likely, respectively). The findings regarding Braden Scale risk categories show a parallel between increased PrI risk often being associated with low Braden Mobility subscale scores and are similar to the findings of Hyun and colleagues,^28^ who discovered the presence of lower Braden total scores (higher risk) among individuals with a PrI. The lower the Braden risk (higher Braden Scale total scores), the more likely a resident is to be capable of moving independently and even walking on and off the clinical unit. Thus, the presence of more low-risk individuals in cluster 3 strongly supports the pattern of equal lying and upright time among these residents.

Two additional theoretically important independent resident characteristics (NH, age) predicted membership in clusters 2 and 3. Nursing home location varied by geographic area, which may have led to differences in the populations by NH. Age was a determining factor in cluster membership; the likelihood of membership in cluster 2 decreased by 2% with each year of advancing age, whereas cluster 3 increased by 2%. Advancing age is commonly understood as an influencer of changes in mobility level.

Dementia and obesity did not emerge as significant predictors of movement patterns after accounting for Braden Scale risk, age, and NH, as found in Table 3. Tables 4 and 5 provide insight into differences in dementia and obesity across clusters (Table 4) and within clusters (Table 5). One-way analysis of variance with post hoc tests (Table 4) showed that most differences between the three clusters within dementia or obesity were significantly associated with either Braden Scale risk categories, mean Braden score, age (dementia), or race (obesity).

Pairwise comparison of residents with dementia and obesity within clusters, as found in Table 5, showed few significant differences. One notable difference between the diagnoses was related to race. Black women have the highest rates of obesity or being overweight when compared with other races according to the US Department of Health and Human Services Office of Minority Health.^29^ Because obesity limits capacity for movement and is considered a risk factor in PrI development, exploration of potential differences in movement among residents of various races with obesity is merited. Future research focusing on how the multiple factors of age, race, and PrI risk interact to influence movement patterns could also prove helpful when designing new strategies to better facilitate movement and enhance PrI prevention.

Significantly lower total upright frequency per hour for residents with dementia in cluster 2 is potentially explained by the tendency for some residents with dementia to remain stationary by sitting in bed or a chair, in contrast to residents with obesity who often avoid movements that may trigger joint and back pain. The greater PrI risk reflected in significantly lower mean Braden scores of residents with dementia in both clusters 2 and 3 may indicate disease progression to a stage of illness when the resident would be prone to fewer position frequency changes. The metrics used to define homogeneous movement patterns (clusters) that were determined in daily units based on sensor recordings may have contributed to the absence of differences. This approach has the potential to diminish the ability to detect different movement patterns for residents with dementia and obesity during different shifts of the day. For example, residents with dementia may experience sundowning that typically occurs in late afternoon or evening; factors such as this that can produce variations in movement patterns that may not have been detected because of the dependence on time of day.

The continued growth in the number of residents with dementia and obesity in NHs heightens the importance of exploring resident characteristics in relation to PrI incidence. Nursing home residents rely on self- or nurse-assisted repositioning/movement as a key strategy for prevention of PrI development. Alterations in mobility are known to place residents with dementia or obesity at greater PrI risk. Thus, the results from examining their movement patterns provide valuable information to help advance knowledge about clinical practices that may improve PrI prevention.

### Limitations

The analyses conducted for this study relied on secondary data from the TEAM-UP clinical trial. The collection of resident data using a wireless sensor captured movement data every 10 seconds, 24 hours a day, while a sensor was worn. The daily metrics used in the current study’s analyses required the omission of sensor data for partial days. In addition, the daily sensor metrics were based on a varied number of days of observations (1–28 days; mean, 16 days) with an average of 22 to 24 hours per day. This analysis approach could influence study findings even though partial days comprised a small amount of data and the mean number of days per resident was 16.

There are limitations to K-means clustering that also may have influenced study results. This method does not produce an optimal number of clusters (ie, value for *k*). Instead, the choice of *k* required an evaluation of a range of cluster solutions that might be useful. Each choice of *k* produced different subgroups. The final choice of three clusters was based on clinical evaluation comparing the competing solutions to identify the one most relevant for this study.

Generalizability of secondary data analysis findings may be limited by the absence of PrI development during the TEAM-UP trial.^19,20^ The TEAM-UP findings extended evidence about repositioning residents using high-density foam mattresses at 2-, 3-, and 4-hour intervals to a 4-week study period (which was longer than prior studies) with no PrI development. These findings advanced PrI prevention knowledge by demonstrating that most residents could be repositioned at a 3- or 4-hour interval and still prevent PrIs. The current study associated movement patterns with the resident’s history of a healed PrI but could not directly associate movement patterns with the development of PrI. In this case, a history of a healed PrI is believed to be a reasonable proxy because these residents are more likely to develop a second PrI. Further validation directly associating movement patterns and PrI is recommended in future research examining repositioning in which PrIs occur.

## CONCLUSIONS

This study identified three clusters of movement patterns. The combined use of dementia diagnoses and BIMS score as indicators of dementia provided a broader cohort of those in varied stages of dementia than has been explored in prior research. Study results provide initial understanding of the influence of dementia and obesity on movements, with the potential to improve repositioning protocols for more effective PrI prevention. Lying and upright position frequencies and durations provide foundational knowledge needed to tailor PrI prevention interventions despite few significant differences in movement patterns for residents living with dementia or obesity. Future research should build on these findings by exploring the individual resident’s frequency or duration of prolonged or sustained movement events in comparison with transient or episodic movement events to explain which movement behaviors are effective in reducing PrI development.
